# Transfer of the longevity-associated variant of BPIFB4 gene rejuvenates immune system and vasculature by a reduction of CD38^+^ macrophages and NAD^+^ decline

**DOI:** 10.1038/s41419-022-04535-z

**Published:** 2022-01-27

**Authors:** Elena Ciaglia, Valentina Lopardo, Francesco Montella, Albino Carrizzo, Paola Di Pietro, Marco Malavolta, Robertina Giacconi, Fiorenza Orlando, Monica Cattaneo, Paolo Madeddu, Carmine Vecchione, Annibale Alessandro Puca

**Affiliations:** 1 Department of Medicine, Surgery and Dentistry “Scuola Medica Salernitana”, University of Salerno, Via Salvatore Allende, 84081 Baronissi Salerno, Italy; 2 Department of AngioCardioNeurology, IRCCS Neuromed, Pozzilli, 86077 Isernia, Italy; 3 Advanced Technology Center for Aging Research, Scientific Technological Area, IRCCS INRCA, 60121 Ancona, Italy; 4 Cardiovascular Research Unit, IRCCS MultiMedica, 20138 Milan, Italy; 5 Bristol Medical School (Translational Health Sciences), Bristol Heart Institute, University of Bristol, Bristol, UK

**Keywords:** Senescence, Immunological disorders, Inflammatory diseases

## Abstract

As we age, our body experiences chronic, systemic inflammation contributing to the morbidity and mortality of the elderly. The senescent immune system has been described to have a causal role in driving systemic aging and therefore may represent a key therapeutic target to prevent pathological consequences associated with aging and extend a healthy lifespan. Previous studies from our group associated a polymorphic haplotype variant in the BPIFB4 gene (LAV-BPIFB4) with exceptional longevity. Transfer of the LAV-BPIFB4 in preclinical models halted the progression of cardiovascular diseases (CVDs) and frailty by counterbalancing chronic inflammation. In the present study, we aimed to delineate the action of systemic adeno-associated viral vector-mediated LAV-BPIFB4 gene transfer (AAV-LAV-BPIFB4) on the deleterious age-related changes of the immune system and thereby the senescence-associated events occurring in C57BL/6J mice aged 26 months. Our in vivo data showed that 26-months-old mice had a higher frequency of CD45^+^SA-beta Gal^+^ immune cells in peripheral blood than young (4-months-old) C57BL/6J mice. Notably, AAV-LAV-BPIFB4 gene transfer in aged mice reduced the pool of peripheral immunosenescent cells that were shown to be enriched in the spleen. In addition, the proper tuning of the immune secretory phenotype (IL1β^low^, IL6^low^, IL10^high^) associated with a significant reduction in SA-beta Gal-positive area of aorta from AAV-LAV treated mice. At the functional level, the reduction of senescence-associated inflammation ensured sustained NAD^+^ levels in the plasma of AAV-LAV-BPIFB4 old mice by preventing the NADase CD38 increase in F4/80+ tissue-resident macrophages and Ly6C^high^ pro-inflammatory monocytes of the spleen and bone marrow. Finally, to validate the clinical implication of our findings, we showed that Long-living-individuals (LLIs, >95 years), which delay CVDs onset, especially if LAV-carriers, were characterized by high NAD^+^ levels. In conclusion, the new senotherapeutic action of LAV-BPIFB4 may offer a valuable therapeutic tool to control aging and reduce the burden of its pathophysiological disorders, such as CVDs.

## Introduction

Most people (80%) aged ≥80 years are frail and suffer from cardiovascular, metabolic, neurodegenerative disease, and cancer making aging the most important risk factor for late-onset diseases [1]. During aging, there is a progressive lack of cellular homeostasis that make organs less efficient and more susceptible to stressors. An example is the age-related decline of the immune system (termed immunesenescence) that becomes less efficient in surveilling and protecting the organism from inflammatory conditions (inflammaging), thus contributing to the organism ageing [2].

Looking for causal factors in driving aging, the level of NAD^+^, the *well-known* nucleotide regulating cellular homeostasis [3], decreased during aging and in progeroid states, contributing to metabolic dysfunction and a decline in overall fitness [3–7]. Recently, the contribution of the senescent immune system to systemic aging has emerged. Indeed, immune cells undergoing senescence, that is, expressing increased levels of p16^INK4α^, p21^CIP1^, and SA-beta Gal activity and showing a peculiar senescence-associated secretory phenotype (SASP), can drive the loss of tissue homeostasis, damage, and age-associated changes in several peripheral organs, thus contributing to reduced lifespan [8]. A possible mechanistic link among senescent cells (SnC), inflammation, and the decline of NAD^+^ in vivo during aging was recently identified in the NAD glycohydrolase CD38 [9–11]. Cellular senescence trigger inflammaging and consequently CD38^+^ inflammatory cells that lead to NAD^+^ decline.

Accordingly, Sayed et al. recently reported that age-related chronic inflammation can predict vascular aging and exceptional longevity [12]. This evidence is prompting the scientific community to explore new practices and interventions to target the deleterious circuit linking senescence, immune responses, and aging and steer individuals toward a healthier life.

Previous work from our group identified a longevity-associated variant (LAV) of a 4 missense SNPs haplotype in BPIFB4 gene associated in homozygosity with exceptional longevity in three independent populations [13, 14]. BPIFB4 is a secreted protein, its levels are increased in serum of long-living individuals [15], and high BPIFB4 levels classify their health status [16, 17]. Furthermore, a prognostic significance in vascular pathology (i.e., atherosclerotic patients) [18] and recently in COVID-19 severity, has been also conferred to its circulating levels [19]. Likewise, homozygous LAV carriers have higher circulating BPIFB4 levels and showed a huge increase in endothelial nitric oxisynthase (eNOS) activation in peripheral blood mononuclear cells (PBMCs) [16, 20]. From a functional point of view, LAV-BPIFB4 was able to improve protein and ribosomal biogenesis, processes lost during aging, and restore favorable proteostatis [20]. Furthermore, in vivo, LAV-BPIFB4 gene transfer rescued endothelial and eNOS dysfunction of aged mice [13], reduced atherosclerosis and inflammation in ApoE^−/−^ mice [18], rescued heart function in a model of diabetic cardiopathy [21], and reduced frailty in old mice [22]. Concerning the immune compartment, LAV-BPIFB4 showed the capability to drive a macrophage-polarizing effect toward a pro-resolving M2 phenotype in atherosclerotic patients [18] and to redirect peripheral monocyte differentiation into regulatory dendritic cells that can counteract the low-grade chronic inflammatory condition [23].

However, if the observed protective effect of LAV-BPIFB4 may be related to a reduction of the burden of senescent immune cells (SnC) in vivo remain an unsolved question.

## Results

### LAV-BPIFB4 regulates the accumulation of immunosenescent cells in aged mice

We started to monitor the effects of systemic adeno-associated viral vector-mediated LAV-BPIFB4 gene transfer on the immune dynamics in old mice by an extensive flow cytometric approach in peripheral blood and lymphoid tissues (bone marrow and spleen).

To this end, eight C57BL/6J male mice aged 26 months were assigned to two experimental groups: a treatment group, receiving a 60 days intravenous injection (i.v. into the tail vein) of 1*10^14^ viral particles of AAV-LAV-BPIFB4 (*N* = 4 mice), and a control group (AAV-GFP; *N* = 4 mice) receiving an identical injection of AAV-GFP. On the sixtieth day since the beginning of the infection, the senescence-associated β-galactosidase (SA-β-gal) substrate has been used to identify CD45^+^ and CD11b^+^ myeloid cells in freshly isolated PBMCs, splenocytes, and bone marrow (BM)-derived cells to determine cellular senescence in these cells. As control, *N* = 5 young (4-months-old) C57BL/6J male mice were also analyzed.

**Fig. 1:**
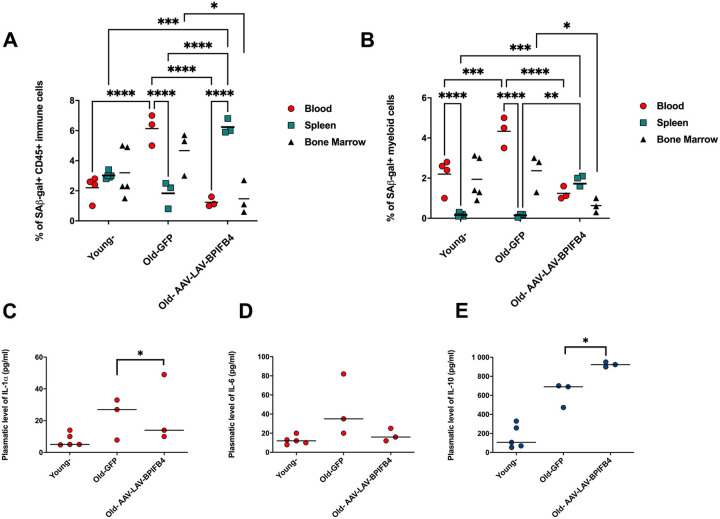
Immunoregulatory effects of AAV-LAV-BPIFB4 infection on cellular senescence in murine blood, spleen, and bone marrow. **A** CD45^+^ immune cells and **B** CD11b^+^ myeloid cells in freshly harvested PBMCs (from *n* = 4 young mice; *n* = 3 Old-GFP mice; *n* = 3 Old-AAV-LAV-BPIFB4), splenocytes (from *n* = 5 young mice; *n* = 3 Old-GFP mice; *n* = 3 Old-AAV-LAV-BPIFB4) and bone marrow-derived cells (from *n* = 4 young mice; *n* = 3 Old-GFP mice; *n* = 3 Old-AAV-LAV-BPIFB4) were assayed by flow cytometry to determine cellular senescence through the Saβ-galactosidase staining. In both panels, the percentage of Saβ-gal expression in gated compartments is shown. Individual values are expressed together with mean. (*P < 0.05, **P < 0.01, ***P < 0.001, ****P < 0.0001). Statistical evaluation was carried out by 2-way ANOVA corrected for Tukey’s multiple comparisons test (GraphPad® Prism). Plasmatic levels of **C** IL-1α, **D** IL-6 and **E** IL-10 were considered to delineate the SASP profile in *n* = 5 young mice, *n* = 3 Old-GFP mice, and *n* = 3 Old-AAV-LAV-BPIFB4 mice. Pairwise comparisons statistically significant are indicated and individual values are expressed together with mean (*P < 0.05, **P < 0.01, ***P < 0.001, ****P < 0.0001). Statistical evaluation was carried out by ordinary one-way ANOVA corrected for Dunnett’s multiple comparisons test (GraphPad® Prism).

As expected, in aged mice we monitored an increase in SA-beta Gal activity in peripheral blood mononuclear cells (Fig. 1A). This increase is most significant in CD11b^+^ myeloid cells (Fig. 1B), without affecting neither CD3^+^ T nor NK1.1^+^Natural Killer (NK) cell compartment (Supplementary Fig. 1). Notably, 60 days of AAV-LAV-BPIFB4 infection resulted in a significant decrease in senescent pool of peripheral immune cells and a concomitant enrichment of senescent cells in the spleen of AAV-LAV-BPIFB4 mice compared to old-GFP-mice.

Accordingly, while the level of some circulating SASP factors (IL-1α, IL-6, and IL-10) was increased in the plasma of aged mice (Old-GFP) compared to young wild-type mice, in AAV-LAV-BPIFB4 mice the plasma level of IL-1α was significantly lower than aged mice, almost comparable to young mice (Fig. 1C). In addition, even though it failed to reach a statistical significance, there was a similar trend for the increased IL-6 in plasma of aged mice and its reduction after AAV-LAV-BPIFB4 infection (Fig. 1D). As regards as IL-10 immunomodulatory factor, it was increased with age mainly to counterbalance an inflammatory background, and the AAV-LAV-BPIFB4 infection further increased its level, consistent with an anti-inflammatory action (Fig. 1E).

### LAV-BPIFB4 counteracts the senescence burden in aged-aorta and NAD + decline

**Fig. 2:**
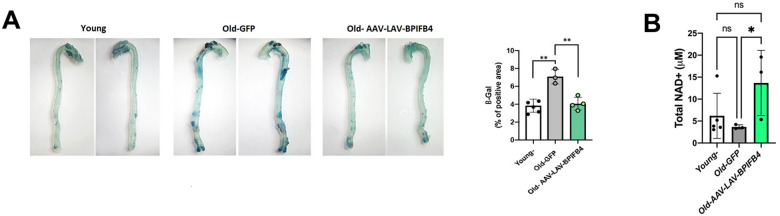
AAV-LAV-BPIFB4 routes the reduction of senescence in aged-aorta and NAD^+^ plasma levels increase. **A** Freshly isolated aortas from mice were stained for Saβ-gal. On the left, representative photographs (*n* = 2 young, *n* = 2 Old-GFP, and *n* = *2* Old-AAV-LAV-BPIFB4 *mice*) of Saβ-gal-stained aortas are shown. On the right, the relative densitometric analysis (*n* = *5* young mice; *n* = 3 Old-GFP mice; *n* = 4 Old-AAV-LAV-BPIFB4 mice) shows the percentage of positive areas. Individual values are expressed as mean ± SD. Pairwise comparisons statistically significant are indicated (**P < 0.01). Statistical evaluation was carried out by ordinary one-way ANOVA corrected for Dunnett’s multiple comparisons test (GraphPad® Prism). **B** Bar graph shows the plasmatic levels of total NAD + in *n* = 5 young mice, *n* = *3* Old-GFP mice, and *n* = *3* Old-AAV-LAV-BPIFB4 mice. Results from each treatment group are expressed as mean ± SD. Pairwise comparisons statistically significant are indicated (*P < 0.05). Statistical evaluation was carried out by ordinary one-way ANOVA and uncorrected Fisher’s LSD test for post-hoc analysis (GraphPad® Prism).

Notably, the senescence observed in immune cells can be sufficient to transfer and/or sustain senescence in a paracrine manner, causing SASP-mediated damage in other tissues [8, 11]. To prove this effect, we next quantified the senescence also in the aorta from young, old-GFP- and AAV-LAV-aged mice. As expected, SA-beta Gal staining of the aorta from aged mice resulted in a great induction of senescence-related immunoreactivity compared to that from young mice; on the contrary, AAV-LAV treated mice showed a significant reduction in SA-beta Gal-positive area compared to the aorta from aged mice (Fig. 2A), probably for a lower inflammatory burden. According to this indirect mechanism, we noticed a huge inhibition of p38 activity, a well-known signaling of SASP induction and maintenance [24], in mesenteric arteries explanted from AAV-LAV-BPIFB4 mice compared to old mice (Supplementary Fig. 2). Further, the herein relative increase in BPIFB4 expression level (Supplementary Fig. 2) confirmed, once again that, after liver transduction of viral vector [18], LAV-BPIFB4 was upregulated in peripheral tissues, mainly through an uptake of circulating protein [18].

From a functional point of view, senescence-associated chronic sterile inflammation in aging has been recently associated with a NAD^+^ decline and so to disease state. This led us to analyze NAD^+^ plasma level in control young mice, and in both AAV-GFP- and AAV-LAV-old mice. Our data documented a decrement in NAD^+^ levels during the aging process in vivo; as expected the AAV-LAV-BPIFB4 treatment promoted a reversal of NAD^+^ decline (Fig. 2B).

### LAV-BPIFB4 affects the NK and macrophage cells activity in the spleen of aged mice and reduces the frequency of CD38^+^ immune cells

**Fig. 3:**
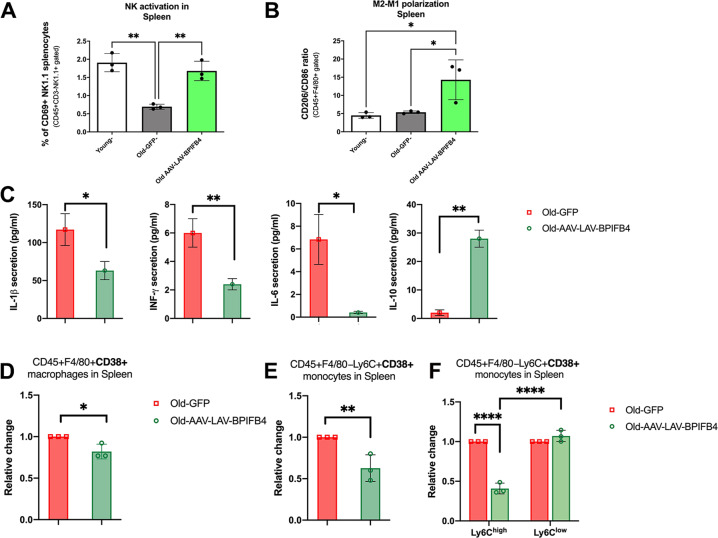
Senescence profile of spleen-resident immune cells and catabolic activity of AAV-LAV-BPIFB4 infection. **A** Flow cytometric quantification of the percentage of gated CD69^+^NK1.1^+^ among freshly isolated CD45^+^CD3-NK1.1^+^ splenocytes in *n* = *3* Young, *n* = *3* Old-GFP, and *n* = *3* Old-LAV-BPIFB4 mice. **B** Flow cytometric quantification of the CD206^+^/CD86^+^ ratio (indicative of the macrophage polarization into a M2 or M1 macrophage-like phenotype) among total CD11b^+^F4/80^+^ splenocytes in *n* = 3 Young, *n* = *3* Old-GFP and *n* = *3* Old-LAV-BPIFB4 mice. Results from each treatment group are expressed as mean ± SD. Pairwise comparisons statistically significant are indicated (*P < 0.05, **P < 0.01). Statistical evaluation was carried out by unpaired *t*-test (GraphPad® Prism)**. C** ELISA quantification of inflammatory cytokines levels in splenocytes conditioned media of *n* = *3* Old-AAV-LAV-BPIFB4 mice as against those of *n* = 3 Old-GFP mice (*P < 0.05, **P < 0.01). Results are expressed as mean ± SD. Pairwise comparisons statistically significant are indicated (*P < 0.05, **P < 0.01). Statistical evaluation was carried out by ordinary one-way ANOVA corrected for Tukey’s multiple comparisons test (GraphPad® Prism). **D** Histogram shows the amount of CD45^+^F4/80^+^ CD38^+^ spleen-resident macrophages in *n* = 3 aged mice infected with AAV-LAV-BPIFB4 compared to *n* = 3 mice with GFP infection (*P < 0.05). Results are expressed as mean ± SD in terms of relative change to maximize the effect compared to their absolute percentage values (Supplementary Fig. 4). Pairwise comparisons statistically significant are indicated (*P < 0.05). Statistical evaluation was carried out by ordinary one-way ANOVA corrected for Tukey’s multiple comparisons test (GraphPad® Prism). **E** Bar graph shows the amount of CD45^+^F4/80-Ly6C^+^CD38^+^ spleen-resident monocytes in *n* = 3 aged mice infected with AAV-LAV-BPIFB4 compared to *n* = 3 GFP-infected mice (**P < 0.01) expressed as mean ± SD in terms of relative change. Pairwise comparisons statistically significant are indicated (**P < 0.01). Statistical evaluation was carried out by ordinary one-way ANOVA corrected for Tukey’s multiple comparisons test (GraphPad® Prism). **F** Frequency of CD45^+^F4/80-Ly6C^+^CD38^+^ spleen-resident monocytes stratified into Ly6C^high^ and Ly6C^low^ from *n* = 3 Old-AAV-LAV-BPIFB4 mice vs *n* = 3 Old-GFP mice is reported (****P < 0.001). Results are expressed as mean ± SD. Pairwise comparisons statistically significant are indicated (****P < 0.0001). Statistical evaluation was carried out by ordinary one-way ANOVA corrected for Tukey’s multiple comparisons test (GraphPad® Prism).

Recently a CD38-dependent NADase activity in the immune cell compartment, especially in tissue-resident pro-inflammatory M1-like macrophages of aged tissues, has been proposed as a mechanism of NAD^+^ decline [10]. The accumulation of CD38 in vivo is induced by SASP in several tissues, as reported for liver, spleen, pancreas, and white-adipose tissue (WAT) [9]. As the AAV-LAV-BPIFB4 infection was able to remove from the blood the circulating senescent cells by redirecting them to the spleen and rescue NAD^+^ level, we asked if the effects of AAV-LAV-BPIFB4 might be related to the CD38 modulation. At first, we moved to clarify the nature of the enrichment of senescent cells in the spleen, the main haemocateretic organ, of AAV-LAV-BPIFB4 old mice. As expected, the aged mice have less active/resident NK cells than young mice (Fig. 3A), probably because of the age-associated immune decline. The AAV-LAV-BPIFB4 treatment significantly increases the percentage of active NK1.1^+^CD69^+^ cells (Fig. 3A) among the total splenocytes (Fig. 3A) and restores their proliferative index upon in vitro treatment with Cell TransAct™ Stimulatory Reagent (Supplementary Fig 3). This might suggest a rescue of the immunosenescence through a sustained activity of cytotoxic NK cells to properly eliminate immunosenescent cells. In support of this, we also reported the degree of M2/M1 polarization in the spleen in all experimental groups (Fig. 3B). As shown in Fig. 3B, there are no differences in CD206^+^/CD86^+^ ratio between young and old mice; but the AAV-LAV-BPIFB4 infection determined a significant increase in M2 pro-resolving macrophage, as reported elsewhere [18]. The putative catabolic activity was also corroborated by the reduced inflammatory milieu found in the spleen of AAV-LAV-BPIFB4- with respect to AAV-GFP-old mice (IL1β^low^, IL6^low^, IFN-γ^low^ IL10^high^) (Fig. 3C).

Consistent with a more efficient senotherapeutic process, long-term AAV-LAV-BPIFB4 treatment reduced the accumulation of CD38^+^F4/80^+^ tissue-resident macrophages in the spleen of aged mice (Fig. 3D). Similarly, the frequency of CD38^+^Ly6C+ tissue-resident monocytes (Fig. 3E) and to a better extent than of CD38^+^Ly6C^high^ pro-inflammatory monocytes was reduced after AAV-LAV-BPIFB4 infection, as compared to those of AAV-GFP-aged mice (Fig. 3F).

### The unique microenvironment of the spleen of LAV-BPIFB4 mice skews the phenotype of RAW264.7 macrophages in vitro

**Fig. 4:**
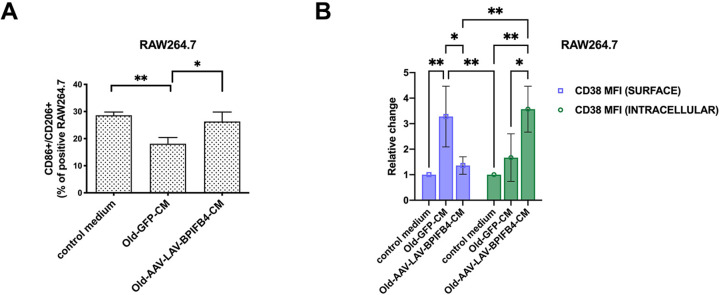
Polarizing effects of AAV-LAV-BPIFB4 splenic milieu on RAW264.7 macrophages in vitro. **A** Analysis of flow cytometry displays the percentage of CD86^+^CD206^+^ M2 macrophages in RAW264.7 cells after 24 h treatment with *Old-GFP* and *Old-AAV-LAV-BPIFB4* splenocytes conditioning media. Results are expressed as mean with SD. Pairwise comparisons statistically significant are indicated (*P < 0.05, **P < 0.01). Statistical evaluation was carried out by ordinary one-way ANOVA corrected for Tukey’s multiple comparisons test (GraphPad® Prism). **B** Histogram shows the mean fluorescent intensity of both surface and intracellular CD38 expression on RAW264.7 cells exposed to splenocytes conditioned media for 24 h. Results are expressed as mean ± SD. Pairwise comparisons statistically significant are indicated (**P < 0.01, **P < 0.01). Statistical evaluation was carried out by ordinary 2-way ANOVA corrected for Šídák’s multiple comparisons test (GraphPad® Prism).

To determine if the peculiar cytokine milieu of the spleen was functional in conditioning other cells in a cell non-autonomous effect, we decided to collect the conditioned medium of the splenocytes of old-GFP mice and old-AAV-LAV-BPIFB4 and to use them to stimulate RAW264.7 macrophage-like cell line in vitro. Indeed, in this experimental setting, while conditioned medium (CM) from AAV-GFP-old mice splenocytes reduced the percentage of M2 (CD86^+^CD206^+^) positive cells in cultured RAW264.7 macrophages, the AAV-LAV-BPIFB4 spleen promoted the M2 phenotype (CD86^+^CD206^+^) (Fig. 4A). Furthermore, while the SASP related factors in the spleen of old-GFP mice (old-GFP-conditioned medium CM) promoted CD38 expression on the surface of RAW264.7, this upregulation of surface CD38 did not occur in RAW264.7 cultured with CM from the spleen of old-AAV-LAV mice. On the contrary, old-AAV-LAV-BPIFB4 CM significantly accumulated the RAW264.7 intracellular expression of CD38 suggesting its proper internalization by old-AAV-LAV-BPIFB4 CM treatment (Fig. 4B).

According to the acquired anti-inflammatory phenotype, we also recorded a slight modulation of inflammatory mediators secreted by RAW264.7 when exposed to the different treatments. While a significant increase of MCP-1, TNF-α, IL-1β, IL-6, IFN- β was induced by CM from the splenocytes of old-GFP mice, a relative reduction when exposed to CM from the old-AAV-LAVBPIFB4-mice splenocytes was documented (Supplementary Fig. 5).

### LAV-BPIFB4 differently affects the pool of CD38^+^ inflammatory cells in the blood and bone marrow of aged mice

**Fig. 5:**
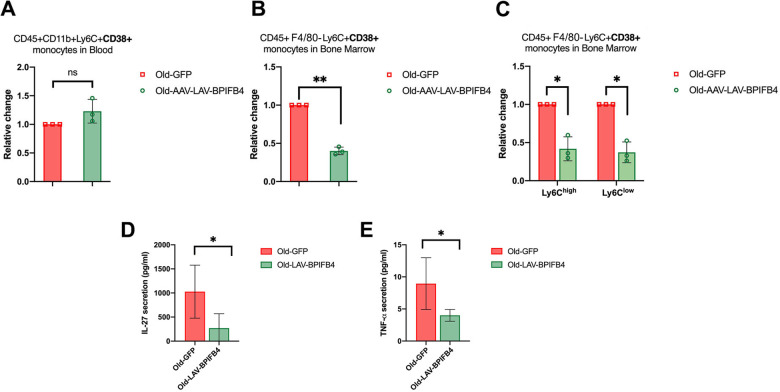
AAV-LAV-BPIFB4 tunes lower CD38 expression in aged blood and bone marrow monocytes shaping an anti-inflammatory microenvironment. Blood and bone marrow monocytes in *n* = 3 Old-GFP mice and *n* = 3 Old-AAV-LAV-BPIFB4 mice were interrogated by flow cytometry gating on CD45^+^F4/80-Ly6C^+^CD38^+^ cells. Panels show **A** circulating monocytes and **B** bone marrow resident monocytes frequency in *n* = 3 Old GFP mice and *n* = 3 Old-AAV-LAV-BPIFB4 mice. **C** Histogram shows the amounts of CD45^+^F4/80-Ly6C^+^CD38^+^ monocytes in bone marrow regarding the stratification in Ly6C^high^ and Ly6C^low^ monocytes (*P < 0.05). Results are expressed as mean ± SD. Pairwise comparisons statistically significant are indicated (ANOVA; *P < 0.05, **P < 0.01). Statistical evaluation was carried out by ordinary one-way ANOVA corrected for Tukey’s multiple comparisons test (GraphPad® Prism). **D** IL-27 and **E** TNF-α secretion levels in bone marrow microenvironment of Old-GFP mice vs Old-AAV-LAV-BPIFB4 mice (*P < 0.05) expressed as mean ± SD. Statistical evaluation was carried out by ordinary one-way ANOVA corrected for Tukey’s multiple comparisons test (GraphPad® Prism).

Being that not only tissue-resident macrophages, but also peripheral and immune precursor cells expressed CD38 [25], we further characterized the frequency of CD38^+^ cells in the blood and bone marrow of AAV-LAV-BPIFB4 infected compared to those of AAV-GFP-aged mice. Of note, we demonstrated no significant effect of AAV-LAV-BPIFB4 treatment in the circulating CD38^+^Ly6C^+^ monocytes (Fig. 5A), while a clear reduction in CD38^+^Ly6C^+^ monocytes (on both Ly6C^high^ and Ly6C^low^ cell subsets) was observed in tissue bone marrow when comparing AAV-LAV-BPIFB4 aged mice to old AAV-GFP-mice (Fig. 5B, C). Accordingly, the bone marrow cytokine milieu of AAV-LAV-BPIFB4 treated mice was mainly characterized by a low level of pro-inflammatory factors, in particular TNF-alpha and reduced levels of IL-27 (Fig. 5D, E).

### LAV-carriers long-living individuals (LLIs) are characterized by high level of NAD^+^

**Fig. 6:**
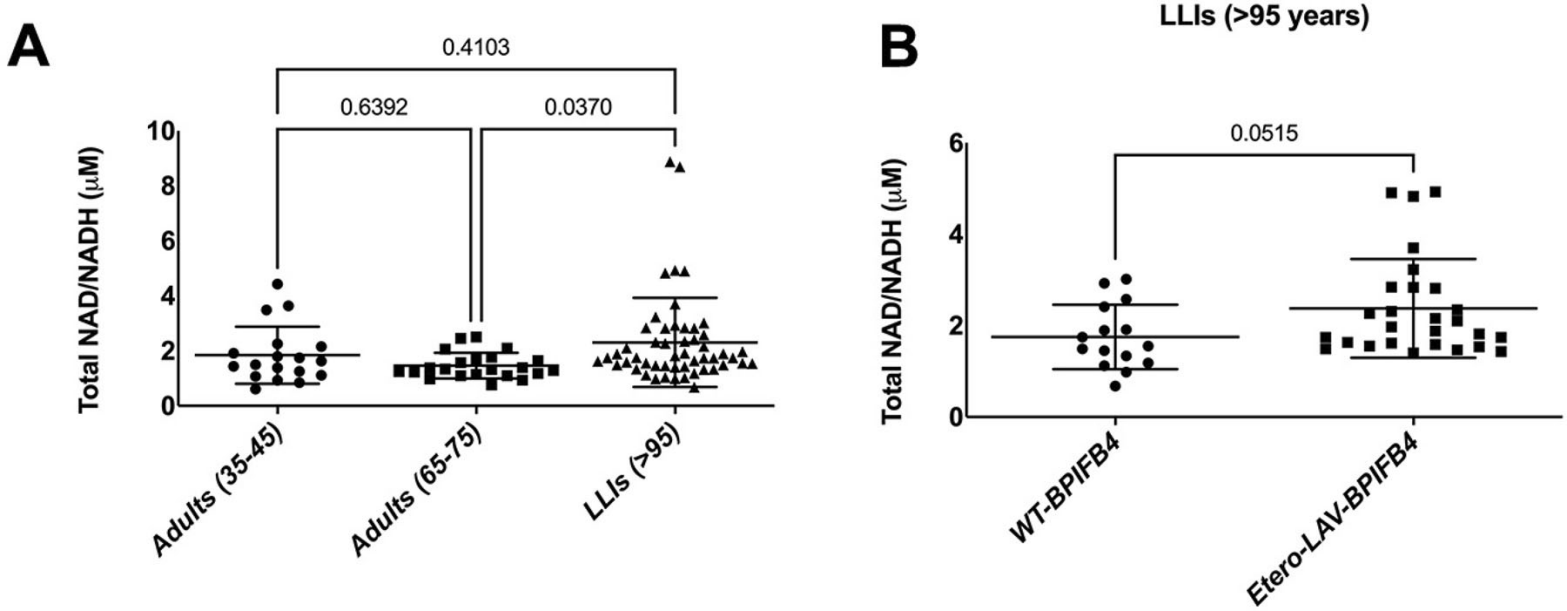
High NAD^+^ circulating levels might protect Long-living individuals. **A** Panel shows the total NAD/NADH levels in plasma from control volunteers of different ages (*n* = 18 adults aged 35–45, and *n* = 22 adults aged 65–75) and LLIs (*n* = 52, aged > 95) expressed as mean ± SD. Individual *P* values are shown. Statistical evaluation was carried out by ordinary one-way ANOVA corrected for Tukey’s multiple comparisons test (GraphPad® Prism). **B** Panel shows total NAD/NADH plasmatic levels following genotype stratification of LLIs in *n* = 15 WT-BPIFB4 carriers and *n* = 27 Etero-LAV-BPIFB4 carriers. Results are expressed as mean ± SD and individual *P* values are shown. Statistical evaluation was carried out by a two-tailed unpaired *t-*test (GraphPad® Prism).

To increase the power of our hypothesis and contemporary give a translational value to the Longevity-associated variant (LAV^−^) of the BPIFB4 gene which may be associated with a reduced senescence burden in vivo, we finally decided to look for NAD^+^ levels as a valuable outcome measure of healthy aging in our cohort of *N* = 52 long-living individuals (LLIs). For these, the analysis of human plasma revealed a statistically significant increase in the accumulation of NAD^+^ levels in LLIs in comparison to old healthy controls (Fig. 6A). Moreover, genotype stratification analysis revealed that LAV carriers (both hetero and homozygous) displayed significantly higher NAD^+^ circulating level, when compared with no-carriers (Fig. 6B). All these may clearly suggest a capability of LLIs and of LAV-carriers to partly contain the deleterious NAD^+^ decline which may fuel their capability to develop more resiliency to age-related diseases.

## Discussion

The inflammatory burden and the decline in immune efficacy experienced by aged organisms are not only the consequence of the aging process but also key drivers in senescence response. As a proof, mouse hematopoietic cells undergoing senescence by genetic approach have shown the ability to transfer aging phenotype to non-lymphoid organs, here causing loss of tissue homeostasis and premature onset of morbidities [8]. This scenario conferred to the peculiar immune signature of the elderly the prognostic value to assess tissue health and predict age-related disease [12]. Taking these aspects into consideration, the pursuit of biomarkers and genetic determinants associated with longevity has become imperative to extend the number of years a person lives in good health.

Here we present exciting new results on the pleiotropic activity of LAV-BPIFB4, a well-known determinant of healthy aging [15–19] and resiliency to develop cardiovascular disease [18, 21, 22]. We describe for the first time the LAV-BPIFB4 efficacy in contrasting immunosenescence, inflammatory cytokine release, aorta senescence, activation of CD38^+^ cells, and NAD^+^ decline in a murine model of advanced ageing. Interestingly, these benefits have a translational value as they are corroborated by the high levels of NAD^+^ we found in Long-living-individuals (LLIs, >95 years), especially if LAV-carriers (Fig. 6), which may help to clarify the association between the health-life expectancy of LLIs and the peculiar genetic profile.

Indeed, central to energy metabolism, NAD^+^ can, directly and indirectly, influence many critical processes for maintaining tissue homeostasis and for healthy aging including DNA repair, chromatin remodeling, and mainly cellular senescence and immune cell functions [3, 26–28]. As consequence, the decline in NAD^+^ levels accompanying chronological aging in both humans and rodents, is one of the major culprits for the development of frailty and age-related metabolic and health decline [3, 29, 30].

Here we provide evidence that the transfer of LAV-BPIFB4 gene can prevent the physiological NAD^+^ depletion through a reduction of the expression of CD38 which decreases tissue NAD^+^ levels through a combination of intracellular NADase and extracellular NMNase activities [25]. Also known to regulate multiple processes in the cells, such as cytokine release [31–33], adhesion and cellular migration to the site of inflammation [34], phagocytosis, and antigen presentation [35, 36], as we age, CD38 protein levels appeared elevated in multiple tissues and organs [3, 25, 29]. On the contrary, the inhibition of CD38 by boosting NAD^+^ levels, can improve glucose and lipid metabolism, by protecting against age- and diet-induced diabetes and obesity [37, 38]. Furthermore, the targeting of CD38-dependent NAD^+^ metabolism has been seen to ameliorate the neuroinflammation associated with the Aβ pathology [39] and to mitigate multiple organ fibrosis [40], other key aging-associated features.

Concerning the mechanism, LAV-BPIFB4 blunted CD38 expression by the reduction of the frequency of senescent immune cells (Fig.1A) and mainly that of CD11b^+^ myeloid cells. Indeed, according to Yousef et al. [8], among most immune cell types CD11b^+^ myeloid cells appeared particularly enriched for senescence marker (p16, p21) in aged wild-type mice and possibly more vulnerable to LAV-BPIFB4 senotherapeutic action in our model.

The high load of immunosenescent cells in the spleen of AAV-LAV-BPIFB4 treated mice would suggest their functional redirecting to the main hemocateretic organ where a clearance of the pool of SnC occurs. Indeed, the higher percentage of active NK1.1^+^CD69^+^ cells (Fig. 3A) and of M2 pro-resolving macrophages (Fig. 3B) corroborates the present hypothesis. As a possible explanation, the reduction of the senescence process in the blood of LAV-BPIFB4-treated mice may downregulate common immune evasion mechanisms of the SnC, thereafter, helping them to become more vulnerable to the cytotoxic attack. This is supported by the recent evidence that SASP can induce senescence-related class Ib murine Qa-2 in autocrine and paracrine manner; this antigen at the same degree of its human HLA-E homolog, can inhibit NK and TCD8^+^ immune responses against SnC [41] contributing to their deleterious persistence. Otherwise, it is conceivable that the AAV-LAV-BPIFB4 infection may induce a differential chemokine pattern of expression, thus regulating the chemotactic response of immune SnC to the spleen. Among these, CXCL12/ stromal cell-derived factor-1 (SDF-1), a ligand for the chemokine receptors CXCR4 is recognized to induce the migration toward the spleen of certain blood cells, such as NKT and endothelial progenitor [42, 43]. This may be consistent with our previous reports showing the participation of SDF-1/CXCR4 axis in most of LAV-BPIFB4 effects [18, 21, 44]. Whatever the mechanism, the reduction of aged-immune cells in LAV-BPIFB4 mice blunted the SASP (Fig. 1C–E) and the deleterious vicious circle affecting adjacent tissues, such as aorta (Fig. 2 and Supplementary Fig. 2) and all kinds of cells exposed to. Likewise, we also demonstrated the peculiar ability of the spleen conditioning medium to properly downregulate CD38 expression on RAW264.7 macrophages (Fig. 4B) in vitro. One interesting question is how CD38 surface expression in RAW264.7 is modulated upon treatment. We postulate that the lower burden of inflammatory mediators of the AAV-LAV-BPIFB4 spleen microenvironment (made up of low levels of IL-1α, IL-6, and IFN-γ with higher IL10 levels) can prevent the CD38 increase on RAW264.7 surface expression, which is in line with the fact that CD38-NADase is primarily regulated by inflammatory nature of the SASP [45].

Another reasonable hypothesis is that peculiar mediators of the host environment may induce CD38 protein internalization [46, 47]. Among these, the leukocyte-shed short sCD31 form, rapidly lost upon TCR-mediated T cell activation in vivo, has been known as the conventional ligand for CD38 receptor [48]. The restoration of a protective T cell reactivity following AAV-LAV-BPIFB4 (Supplementary Fig. 3) led us to speculate that the sCD31 shedding from the surface of activated T cells among AAV-LAV-BPIFB4 splenocytes, might be a signal for the CD38 internalization in RAW264.7 in culture. Even though the hypothesis appears reasonable, the precise nature of this kind of response needs to be carefully dissected.

Furthermore, according to the recent literature which states that CD38^+^ inflammatory cells accumulate mainly in tissues during chronological aging [9], here we reported that LAV-BPIFB4 senotherapeutic action mostly affected the CD38 levels on mono-macrophages of the spleen (Fig. 3) and bone marrow (BM) (Fig. 5B, C), with no effects at blood levels (Fig. 5A). Notably, the reduced accumulation of CD38^+^ inflammatory cells found in BM of AAV-LAV-BPIFB4 old mice is associated with a lower concentration of TNF-alpha and of IL-27 (Fig. 5D, E) in the microenvironment. These cytokines belong to well-known pathways which, when active in aging, mediate aberrancy in hematopoietic stem cells HSC compartment, reduced reconstitution potential, impaired self-renewal, and a pro-inflammatory phenotype [49]. Even though it is still unknown the significance of the accumulation of CD38^+^ inflammatory cells in BM during aging and why these cells have an enzyme to destroy NAD^+^, the results discussed above and the peculiar cytokine milieu may suggest the need for an entry in a resting state during aging, to slow-down those more-energy-consuming mechanisms.

Collectively our results concerning the LAV-BPIFB4 activity in preventing the disruption of NAD^+^ homeostasis, add a new piece of evidence of its association to healthspan and lifespan. However, as with all new studies, numerous questions can be raised. First of all, it would be useful to prove LAV-BPIFB4 senotherapeutic efficacy also in other cell type undergoing senescence (e.g., fibroblasts, hepatocytes, keratinocytes, etc.), which may explain its proven ability to counteract frailty progression in toto in rodents [22]. Also, by reducing immunosenescence, does LAV-BPIFB4 restore the performance of an aged-immune system in terms of an efficient response against pathogens, cancer cells, and the effectiveness of vaccinations? These observations draw attention to our recent findings showing that LLIs showed to be exempted from the pro-inflammatory status typical of inflammaging because of the peculiar monocyte asset capable to polarize toward the anti-inflammatory M2 macrophages in a BPIFB4-dependent manner [15]. This finding together with the inverse correlation between BPIFB4 plasmatic level and the degree of COVID-19 severity [19] really suggest a better tendency to cope with harmful responses during infectious disease or deleterious chronic reactions.

For all the above-mentioned mechanisms, NAD^+^ boosting through both the supplementation of precursors and the inhibition of CD38 is becoming a potential therapeutic strategy to improve the quality of life of aged individuals. However, as its safety and tolerance are yet to be determined in individuals with age-related diseases, here the transfer of LAV-BPIFB4 gene, naturally selected by the evolution, could be a safe and promising therapeutic tool to counteract aging-associated pathologies and/or accelerated aging.

## Methods

### Animal models

Eight C57BL/6J male mice aged 26 months were randomly assigned to two experimental groups: a treatment group (*n* = 4 mice; reported as Old-AAV-LAV BPIFB4 mice) receiving an intravenous injection (i.v. into the tail vein) of 1*10^14^ viral particles of AAV-LAV-BPIFB4 60 days earlier and a control group (*n* = 4 mice reported as Old-GFP mice) receiving an identical injection of AAV-GFP. Five C57BL/6J male mice aged 4 months were analyzed as a young control group (*n* = 5 mice; reported as young mice). The quantification of the results was performed by a second individual who was blind to the genotype of the animal and/or the hypothesis that was being tested for each group.

To reduce the number of animals used for this study, where possible, multiple biochemical, phenotypical, and immune functional analyses were conducted on the same group of mice. The investigators were blinded to group allocation during data collection and analysis

To ensure homogeneity of experimental cohorts, mice from the same F generation were assigned to experimental groups, such that age and weight were matched. The overall wellbeing of each mouse was however the selective inclusion criteria. No specific exclusion criteria were applied. Only if occasional events (e.g., low number of cells, or because of the hemolyzed sample, etc) occurred during the experimental procedures, animals were excluded from the analysis. All criteria were preestablished.

Power analysis has been conducted to determine an adequate number of mice to measure biochemical, phenotypical, and immunological effects in a statistically significant manner. The animal study was reviewed and approved by the Organismo Preposto al Benessere Animale (OPBA, animal care, and health committee) of IRCCS INRCA, Ancona (Italy), and by the Italian Ministry of Health (authorization n. 130/2018-PR).

### Cloning

To obtain BPIFB4 constructs in pAAV2.1 TBG eGFP3 vector, BPIFB4 cDNA was PCR amplified on pRK5 vector with the following primers: AAGCGGCCGCATGCTGCAGCAAAGTGATG for the insertion of *Not*I site at 5′ end; GCAAGCTTTCATGCGCTCAGCACCAAAAG for the insertion of HindIII site at 3′ end. PCR products were purified with Wizard SV Gel and PCR Clean Up System (Promega), digested with *Not*I and *Hin*dIII, and cloned in pAAV2.1 TBG vector replacing the eGFP. All constructs were sequenced for the BPIFB4 gene.

### Vector production and purification

As described previously, vector production and purification of adeno-associated viral vectors were carried out at the AAV vector facility of TIGEM, Italy. In brief, 10-layer cell stacks containing 2.2 × 10^9^ of low passage 293 cells were triple-transfected by calcium phosphate with 1000 µg of pAd helper, that contained the adenovirus E2A, E4, and VA RNA helper genes, 520 µg of pAAV2/9 packaging plasmid with AAV rep and cap genes, and 520 µg of pAAVCis. Medium was changed the following day and cells were harvested 3 days after transfection. Cell lysates were purified by two rounds of cesium chloride centrifugation. For each viral preparation, physical titers (GC ml^−1^) were determined by averaging the titer achieved by dot-blot analysis and by PCR quantification using TaqMan20 (Applied Biosystems, Carlsbad, CA, USA).

### Mice tissue sampling and processing

Spleen, femur, aortas, and blood were obtained from the mice after the sacrifice. Heparinized blood and plasma were collected. Single-cell suspension from bone marrow and spleen were also harvested for analysis by flow cytometry. Aortas were harvested and placed on a wire system for β-galactosidase staining.

Plasma and peripheral blood mononuclear cells (PBMCs) were extracted from the whole blood of mice by Ficoll density gradient (Histopaque®-1077, Sigma-Aldrich). Plasma was employed to estimate the cytokine levels dosage while PBMCs were analyzed by flow cytometry. However, one young mouse’s blood was hemolyzed since it was not enough suitable for PBMCs extraction but quite adequate to dose cytokine levels in plasma.

Spleens were dissociated into single-cell suspension by mechanical digestion. Each spleen was transferred into a sterile 100 mm culture dish containing 10 mL of RPMI-free (Gibco®, ThermoFisher Scientific) supplemented with 2% (v/v) penicillin–streptomycin (Aurogene). Each spleen was pressed using the flat end of a sterile plunger to crush the spleen in circular motions and splenocytes were harvested in a tube to let debris deposit. Cell suspensions were centrifuged at 400 × *g* for 5 min at RT. After discarding the supernatant, cell pellet was resuspended in a 2 mL Red Blood Cell Lysis Buffer and incubated for 4 minutes on ice. To stop the lysis reaction, 10 mL of PBS was added, and the cell suspensions were centrifuged at 400 × *g* for 5 min. After discarding the supernatant, cells were assayed for flow cytometry and cultured to obtain the conditioned medium for cytokines dosage and RAW264.7 cells treatment.

Bone marrow was extracted from the femur through the flushing method on ice. Femurs were cut at both ends with a sterile blade and bone marrow was flushed out by using a 10 cc syringe filled with RPMI-free (Gibco®, ThermoFisher Scientific) supplemented with 2% (v/v) penicillin–streptomycin (Aurogene). Bone marrow-derived cell suspensions were harvested in a tube to let debris deposit. Cell suspension was transferred in a new tube, centrifuged at 1900 rpm for 5 min at RT. After discarding the supernatant, cells were assayed for flow cytometry and cultured to obtain the conditioned medium for cytokines dosage.

### Isolated murine cells treatment

Isolated splenocytes were seeded at 2*10^6^ cells/mL and maintained in culture for 18 h. Following treatment, cell suspensions were collected and centrifuged at 1900 rpm for 5 min at RT. Supernatants were collected and then employed for inflammatory cytokine detection and as conditioned media on RAW264.7 cells for a 24 h treatment.

Bone marrow-derived cells were seeded at 2*10^6^ cells/mL and maintained in culture for 18 h. Following treatment, cell suspensions were collected and filtered through a 100 μm strainer to remove cell clumps. Eluted cells were centrifuged at 1900 rpm for 5 min. Supernatants were collected to perform the cytokine dosage.

### Cell line and culture condition

Properly authenticated RAW264.7 cells (Cat. No.: ATCC® TIB-71™), were grown in humidified incubator at 37 °C and 5% CO_2_ in DMEM (Gibco®, ThermoFisher Scientific) supplemented with 10% (v/v) fetal serum bovine (FBS, Gibco®, ThermoFisher Scientific), 1% (v/v) penicillin–streptomycin (Aurogene), 1% (v/v) MEM non-essential amino acids (MEM NEAA, Gibco®, ThermoFisher Scientific), 1% (v/v) sodium pyruvate (Aurogene). The cell line was tested for mycoplasma contamination before conducting experiments. 400.000 cells/cm^2^ were seeded onto a 12-well plate to be treated for 3 h with 20% of splenocytes conditioned media (previously obtained as above mentioned). After 3 h, RAW264.7 cells pellet was collected and stained for flow cytometry analysis.

### Aortas β-galactosidase staining

To evaluate vascular senescence in the aortic vessels, aortas were fixed for 10 min at room temperature in 2% formaldehyde/0.25% glutaraldehyde solution. Subsequently, vessels were incubated with fresh senescence-β-galactosidase (SA-β-gal) staining solution (Cell SignalingTechnology) at 37 °C for 24 h and then washed three times with PBS before to be acquired with a digital camera Nikon D300s digital camera. Semi-quantitative analysis of SA-β-gal-positive staining was performed with ImageJ software.

### Antibodies and flow cytometry

Single-cell suspensions from murine bone marrow, spleen, PBMCs and RAW264.7 cells were stained with mAb against mouse CD45 (30F11; Miltenyi Biotec; 1:50), mouse CD11b (M1/70.15.11.5; Miltenyi Biotec; 1:50), mouse Ly-6C (REA796; Miltenyi Biotec; 1:50), mouse CD86 (PO3.3; Miltenyi Biotec; 1:10), mouse F4/80 (BM8; Biolegend; 1:10), mouse CD206 (C068C2; Biolegend; 1:50), mouse Qa-2 (695H1-9-9; SONY; 1:50), mouse CD38 (REA616, Miltenyi Biotec; 1:50), mouse SPiDER- β-Gal (SG03-10; Dojindo Molecular Technologies; 1:500), mouse NK1.1 (REA1162, Miltenyi Biotec; 1:50), mouse CD69 (REA937, Miltenyi Biotec; 1:50), mouse CD3 (REA641, Miltenyi Biotec; 1:10). After 20 min incubation at 4 °C in the dark, cells were washed and resuspended in staining buffer for the FACS analysis as previously established [50]. Additional staining for Saβ-galactosidase was performed after the above-mentioned staining and incubated for 15 min at 37 °C in the dark without washing before FACS analysis. For each test, cells were analyzed using FACS Verse Flow Cytometer (BD Biosciences).

### Cytokine detection

Beads-based multiplex ELISA (LEGENDplex, Biolegend, USA) was used to measure cytokine levels (IL-1α, IL-6, IL-10, IL-27, TNF-α) in plasma samples collected from mice and conditioned media from murine spleen, bone marrow, and RAW264.7 cell line. Diluted plasmas and cell culture supernatants were incubated for 2 h with the beads and detection antibodies, followed by 30 min incubation with SA-PE. After washing, beads were resuspended in washing buffer and acquired using a FACS VERSE flow cytometer (BD Biosciences). Data were analyzed with the LEGENDplex Data Analysis Software.

### Western blotting

Mesenteric arteries were harvested and lysed in ice-cold RIPA lysis buffer (50 mM Tris-HCl, 150 mM NaCl, 0.5% Triton X-100, 0.5% deoxycholic acid, 10 mg/mL leupeptin, 2 mM phenylmethylsulfonyl fluoride, and 10 mg/mL aprotinin). After centrifugation (14,000 × *g* for 20 min at 4 °C) in order to remove cell debris, proteins were quantified. About 20 μg of proteins were separated on 12% SDS-PAGE at 90 V for 1 h and at 120 V for 1 h and then transferred to a nitrocellulose membrane. After blocking with 5% nonfat dried milk powder (PanReac AppliChem) in Tris-buffered saline containing 0.1% Tween-20 (TBST) for 1 h at room temperature, the membranes were incubated overnight with the following primary antibodies: anti-p38 MAPK (Biolegend #622401, rabbit pAb 1:500), anti p-p38 (Santa Cruz Biotechnology #7973, rabbit mAb 1:1000), anti-C20orf186 (Abcam #168171 mouse pAb 1:500). Immunodetection of specific proteins was carried out with horseradish peroxidase-conjugated donkey anti-mouse or anti-rabbit IgG (Bio-Rad), using the enhanced chemiluminescence (ECL) system (ThermoFisher Scientific) according to the manufacturer’s instructions and then exposed to X-ray films (ThermoFisher Scientific). Western-blot data were analyzed using Photoshop software to determine the optical density (OD) of the bands. The OD readings of phosphorylated proteins were expressed as a ratio relative to total protein and β-actin.

### NAD/NADH assay

Total NAD and NADH Assay Kit (Colorimetric) (ab186032) was employed to detect total NAD and NADH plasmatic levels in recruited patients following manufacturers’ instructions. 50 μL NAD/NADH reaction mixture were added to 50 μL plasma and incubated at RT up to 2 h. Absorbance was monitored and read with an absorbance plate reader at 460 nm after 1 h and 2 h of incubation.

### Patients’ cohorts

In this study, a group of 104 individuals was recruited*: n* = 40 control volunteers (median age 55, range 35–75) and *n* = 52 long-living individuals (LLIs, median age 97, range 95–99). Control volunteers group was divided in middle-aged adults (*n* = 18; 35–45 years) and old(er) adults (*n* = 22; 65–75 years). These groups were constituted, respectively, by healthy donors and by subjects with no apparent diseases who underwent routine preventive laboratory tests. LLIs were also stratified according to the genotype in WT-BPIFB4 carriers and Etero-LAV-BPIFB4 carriers. For each, venous blood (10 mL) was withdrawn for analyses and detailed anamnesis was collected. LLIs’ whole blood was also collected for genotype determination. For this study, genetic testing for the SNP rs2070235 (p.Ile229Val) on BPIFB4 was assessed in all subjects. From all samples collected, leukocytes were extracted from the whole blood by density gradient (Histopaque®-1077, Sigma-Aldrich) to sort the buffy coat and to extract their genomic DNA (DNeasy kit, Qiagen®, Milano). Then, the DNA was quantified to normalize concentrations run on quantitative polymerase chain reaction (PCR)-Taqman based method.

Plasma was extracted from whole blood by Ficoll density gradient (Histopaque®-1077, Sigma-Aldrich) and used to estimate the NAD/NADH levels. All participants signed an informed consent for the management of personal anamnestic data and blood samples. The study was approved by the IRCCS MultiMedica ethical committee and conducted in accordance with the ethical principles deriving from the Declaration of Helsinki.

### Statistical analysis

In all experiments shown, statistical analysis was performed by using the GraphPad Prism 9.0 software for Windows (GraphPad software). For each type of assay or phenotypic analysis, data obtained from multiple experiments are calculated as mean ± SD and analyzed for statistical significance using appropriate tests. An analysis of variance (ANOVA) for multiple comparison and followed by post-hoc analysis *P* values < 0.05 were considered significant; *P < 0.05, **P < 0.01, and ***P < 0.001.

## Supplementary information

Legends for Supplementary Figures

Supplementary Figure1

Supplementary Figure2

Supplementary Figure3

Supplementary Figure4

Supplementary Figure5

Gating Strategy

Original WB images

reproducibility checklist

## Data Availability

The authors confirm that the data supporting the findings of this study are available within the article and its supplementary materials. Raw data are available from the corresponding authors, [EC and AAP], upon reasonable request.
