# Supplementary material for: Transfer of the longevity-associated variant of BPIFB4 gene rejuvenates immune system and vasculature by a reduction of CD38+ macrophages and NAD+ decline
Source: Cell Death Dis. 2022 Jan 27;13(1):86. doi: 10.1038/s41419-022-04535-z (PMC8792139; doi:10.1038/s41419-022-04535-z)
Supplement: Supplementary file 1 — Legends for Supplementary Figures [file 41419_2022_4535_MOESM1_ESM.docx]

**Transfer of the Longevity associated variant of BPIFB4 gene rejuvenates immune system and vasculature by a reduction of CD38+macrophages and NAD+ decline.**

By:

Elena Ciaglia PhD^1,^*, Valentina Lopardo BS^1^, Francesco Montella PhD^1^, Albino Carrizzo PhD^1,2^, Paola Di Pietro PhD^1^, Marco Malavolta PhD^3^, Robertina Giacconi PhD^3^, Fiorenza Orlando PhD^3^, Monica Cattaneo PhD^4^, Paolo Madeddu MD^4,5^, Carmine Vecchione Professor^1,3^, Annibale Alessandro Puca^1,4,^*  Professor.

1, Department of Medicine, Surgery and Dentistry "Scuola Medica Salernitana", University of Salerno, Via Salvatore Allende, 84081 Baronissi Salerno, Italy;

2, Department of AngioCardioNeurology, IRCCS Neuromed, Pozzilli, 86077 Isernia, Italy;

3, Advanced Technology Center for Aging Research, Scientific Technological Area, IRCCS INRCA, 60121, Ancona, Italy;

4, Cardiovascular Research Unit, IRCCS MultiMedica, 20138 Milan, Italy.

5, Bristol Medical School (Translational Health Sciences), Bristol Heart Institute, University of Bristol, Bristol, United Kingdom

**Running title**: Senotherapeutic action of LAV-BPIFB4 in aged mice

***Correspondence:**

*Annibale Alessandro Puca*, Department of Medicine, Surgery and Dentistry "Scuola Medica Salernitana, University of Salerno, Via Salvatore Allende, 84081 Baronissi Salerno, Italy.

Tel: +39089965235 Fax: +39089969602. E-mail: [apuca@unisa.it](mailto:apuca@unisa.it)

*Elena Ciaglia*, Department of Medicine, Surgery and Dentistry "Scuola Medica Salernitana, University of Salerno, Via Salvatore Allende, 84081 Baronissi Salerno, Italy.

Tel: +39089965115 Fax: +39089969602. E-mail: [eciaglia@unisa.it](mailto:eciaglia@unisa.it)

**Supplementary Figure Legend**

**Supplementary Figure 1. The effect of AAV-LAV-BPIFB4 infection on T and NK cellular senescence in murine blood, spleen and bone-marrow.**

**(A)** CD3+ T cells and **(B)** CD3-NK1.1 Natural Killer (NK) cells in freshly harvested PBMCs, splenocytes and bone marrow-derived cells from *n= 5 young mice, n= 3 Old-GFP mice and n= 3 Old-AAV-LAV-BPIFB4* were assayed by flow cytometry to determine cellular senescence through the Saβ-galactosidase staining. In both panels the percentage of Saβ-gal expression in gated compartments is shown. Individual values are expressed together with mean. (**P<0.01, *** P<0.001). Statistical evaluation was carried out by 2way ANOVA corrected for Tukey's multiple comparisons test (GraphPad® Prism).

**Supplementary Figure 2. The effect of AAV-LAV-BPIFB4 infection on the p38 activation and BPIFB4 expression in mesenteric artery of old mice.**

Representative western blot (left) and densitometric analysis (right) conducted on mesenteric artery lysates. Values are mean ± standard deviation (N= 3). One-way ANOVA followed Tukey’smultiple comparisons test.

**Supplementary Figure 3. Analysis of the proliferation of splenocytes in young and in old mice, infected or not with AAV-LAV-BPIFB4**

Effects on splenocytes proliferation observed by BrdU incorporation ELISA assay. Bar graphs report the percentage ± standard deviation of proliferating cells upon *in vitro* treatment with Cell TransAct™ Stimulatory Reagent for 72h. Statistical analysis by One-way ANOVA followed Tukey’smultiple comparisons test was conducted. Numbers above square brackets show unadjusted LSD P-values.resentative western blot (left)

**Supplementary Figure 4. Percentage of CD38+ monocytes in young and in old mice, infected or not with AAV-LAV-BPIFB4**

**(A)** Bar graph shows the percentage of CD45+F4/80-Ly6C+CD38+ spleen-resident monocytes in *n=3 aged mice infected with AAV-LAV-BPIFB4* compared to *n=3 GFP-infected mice* (*P<0.05) expressed as mean ± SD. **(B)** Frequency of CD45+F4/80-Ly6C+CD38+ spleen-resident monocytes stratified into Ly6C^high^ and Ly6C^low^ from *n=3 Old-AAV-LAV-BPIFB4 mice vs n=3 Old-GFP mice* is reported (**P< 0.01). Statistical evaluation was carried out by ordinary one-way ANOVA corrected for Tukey's multiple comparisons test (GraphPad® Prism).

**Supplementary Figure 5. The effects of AAV-LAV-BPIFB4 splenic milieu on RAW264.7 secretory profile *in vitro*.**

Secretory profile of RAW264.7 cells after 24h treatment with splenocytes conditioned media, as detected by Multiplex ELISA of cell medium. Statistical analysis by two-way ANOVA with Tukey test for multiple comparison was conducted. (p-value ***<0.001).
