# Supplementary figures and images for: Transfer of the longevity-associated variant of BPIFB4 gene rejuvenates immune system and vasculature by a reduction of CD38+ macrophages and NAD+ decline
Source: Cell Death Dis. 2022 Jan 27;13(1):86. doi: 10.1038/s41419-022-04535-z (PMC8792139; doi:10.1038/s41419-022-04535-z)

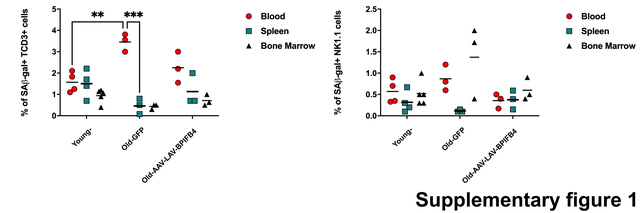

Supplement: Supplementary file 2 — Supplementary Figure1 [file 41419_2022_4535_MOESM2_ESM.jpg]

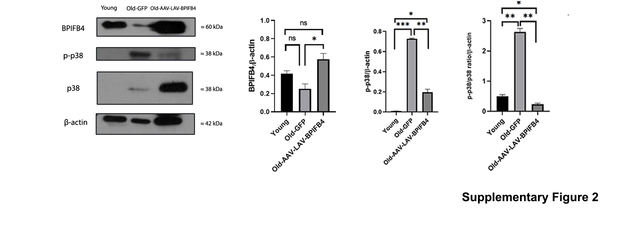

Supplement: Supplementary file 3 — Supplementary Figure2 [file 41419_2022_4535_MOESM3_ESM.jpg]

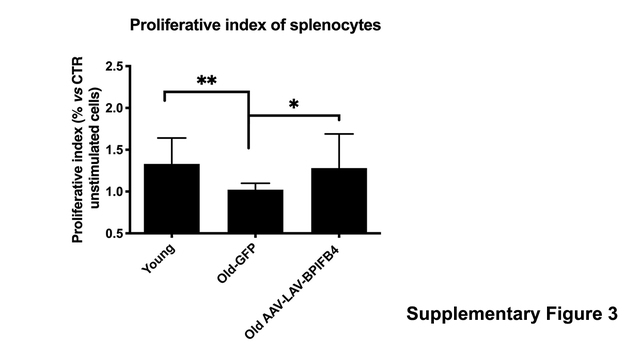

Supplement: Supplementary file 4 — Supplementary Figure3 [file 41419_2022_4535_MOESM4_ESM.jpg]

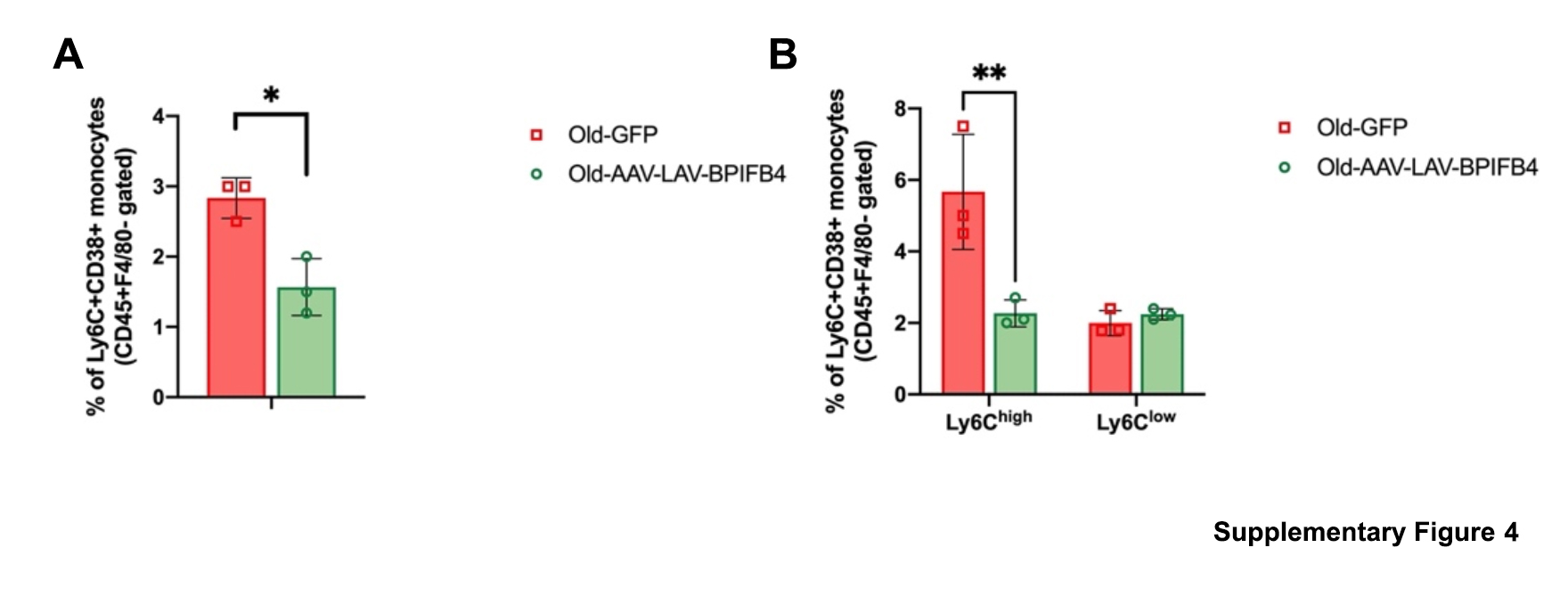

Supplement: Supplementary file 5 — Supplementary Figure4 [file 41419_2022_4535_MOESM5_ESM.jpg]

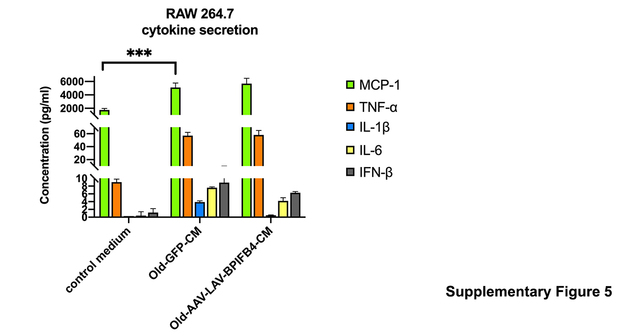

Supplement: Supplementary file 6 — Supplementary Figure5 [file 41419_2022_4535_MOESM6_ESM.jpg]

## Slide 1
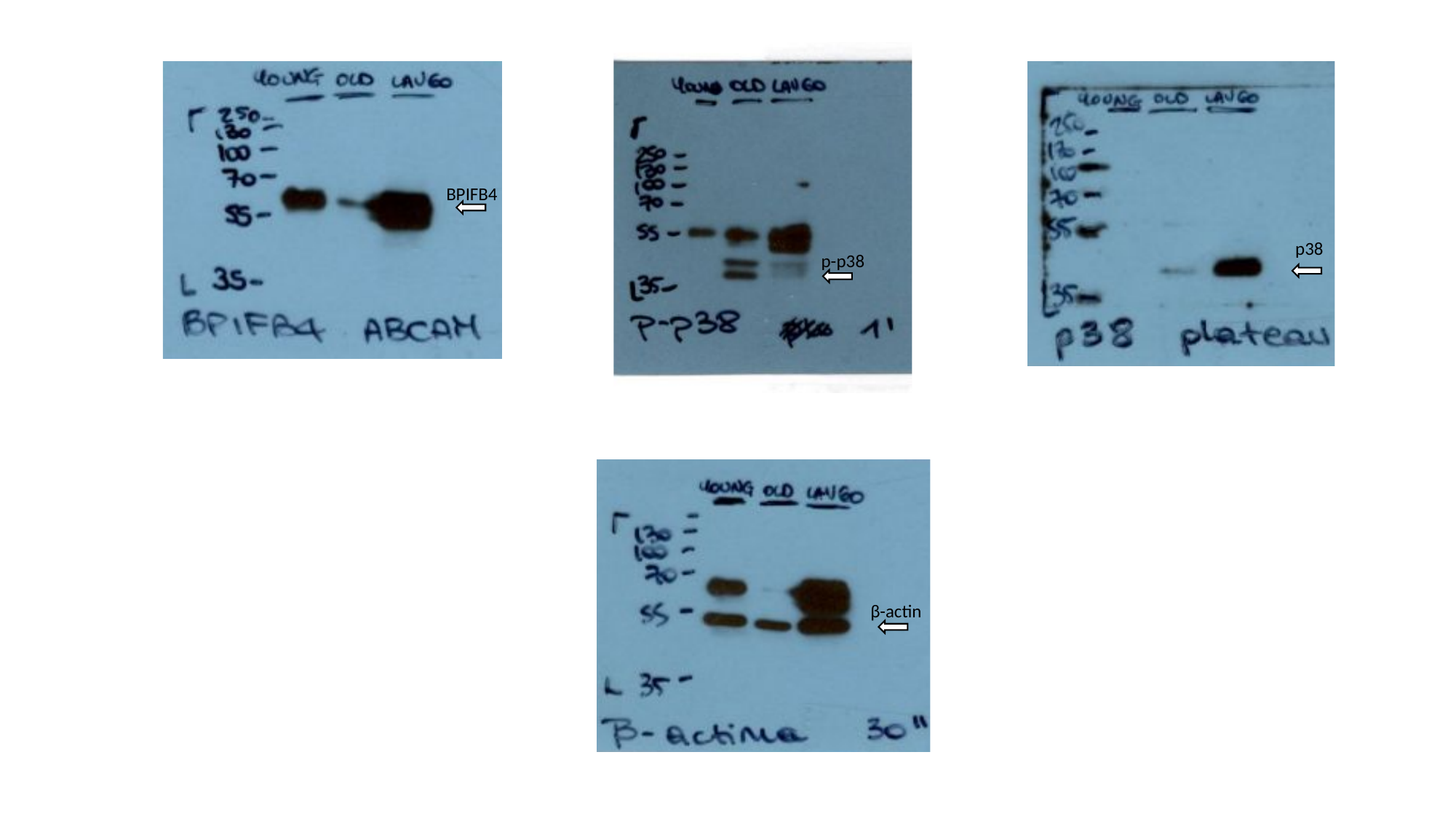

p-p38
BPIFB4
p38
β-actin

Supplement: Supplementary file 8 — Original WB images [file 41419_2022_4535_MOESM8_ESM.pptx]
