# Supplementary material for: Transfer of the longevity-associated variant of BPIFB4 gene rejuvenates immune system and vasculature by a reduction of CD38+ macrophages and NAD+ decline
Source: Cell Death Dis. 2022 Jan 27;13(1):86. doi: 10.1038/s41419-022-04535-z (PMC8792139; doi:10.1038/s41419-022-04535-z)
Supplement: Supplementary file 7 — Gating Strategy [file 41419_2022_4535_MOESM7_ESM.pdf]

“Transfer of the Longevity associated variant of BPIFB4 gene rejuvenates immune system and vasculature by a reduction of CD38+macrophages and NAD+ decline”

GATING STRATEGY

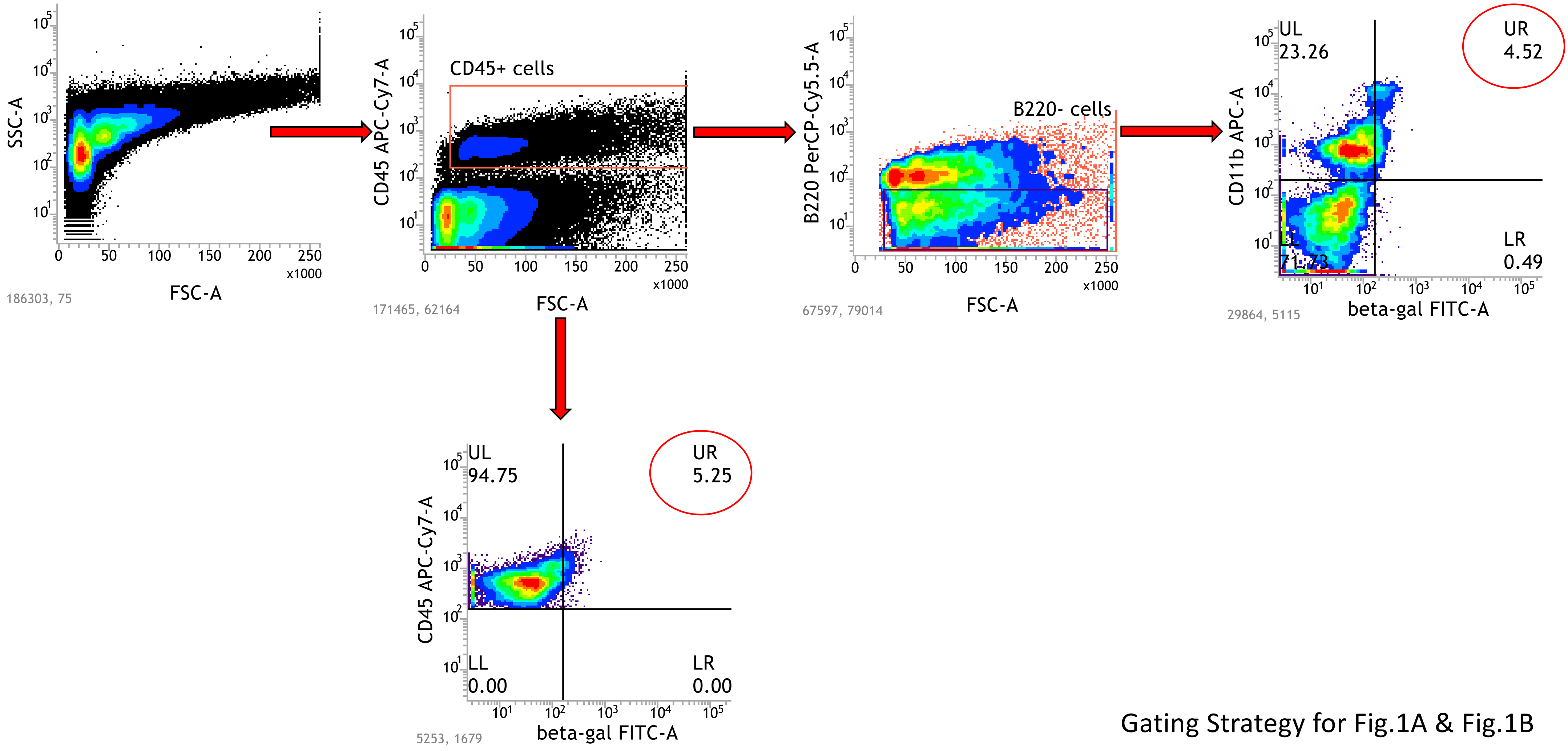

Gating Strategy for Fig.1A & Fig.1B

BLOOD

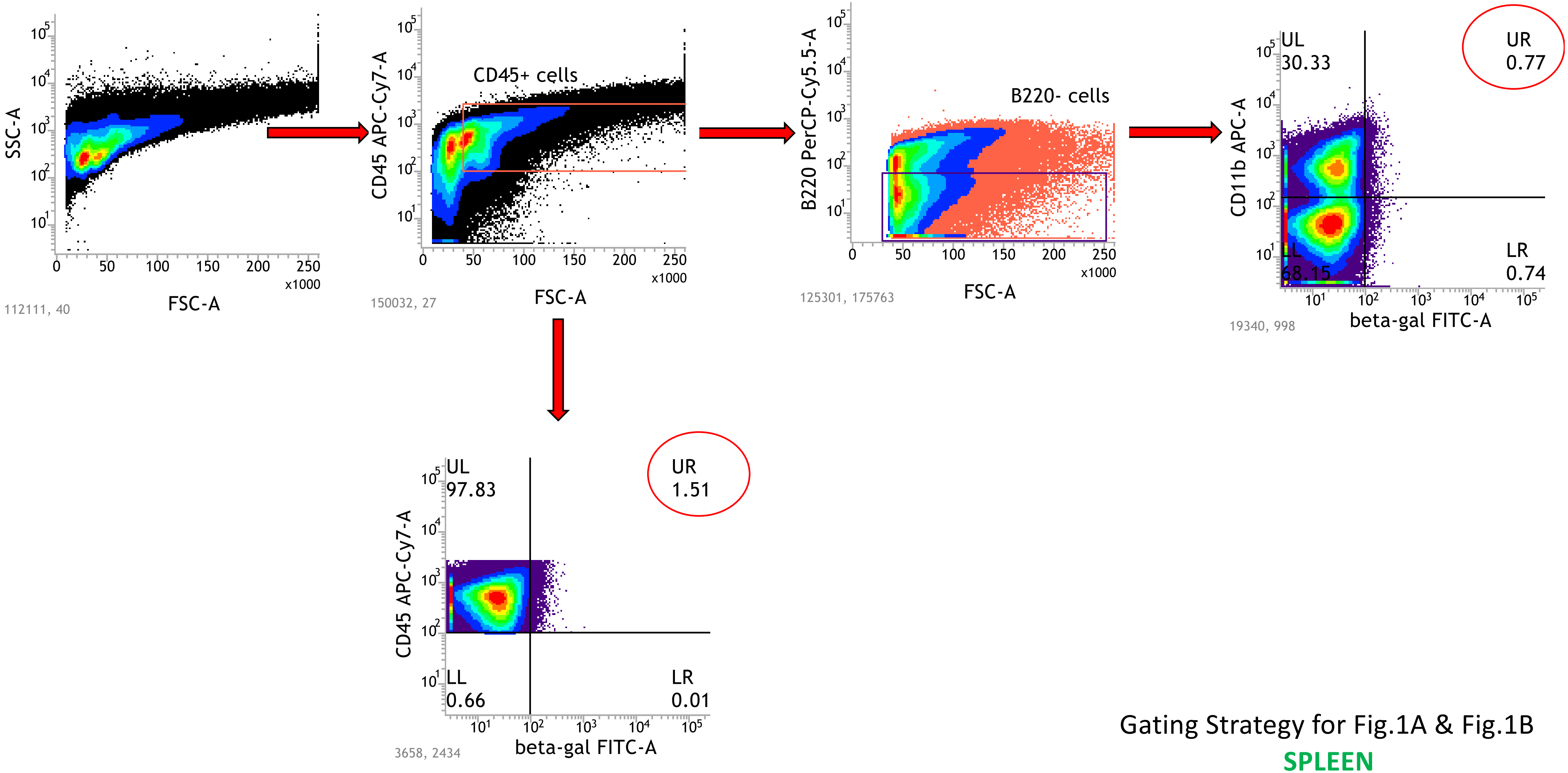

Gating Strategy for Fig.1A & Fig.1B

SPLEEN

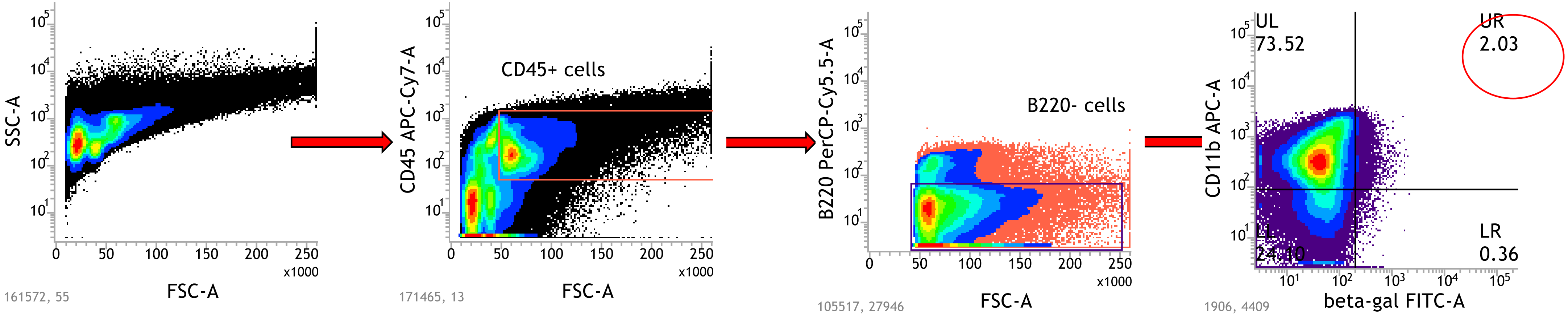

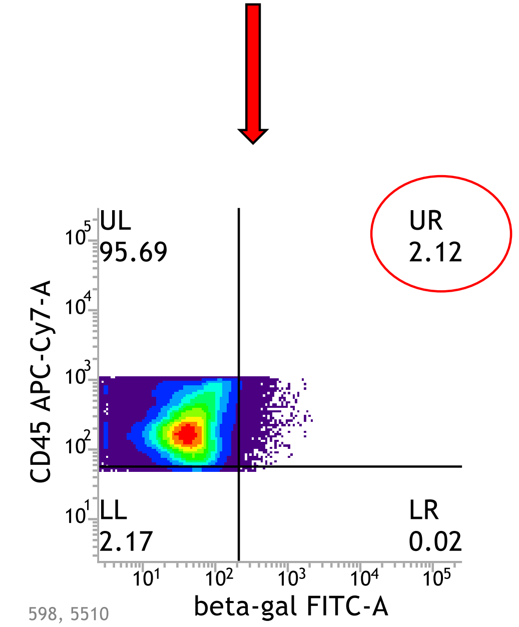

Gating Strategy for Fig.1A & Fig.1B

BONE MARROW

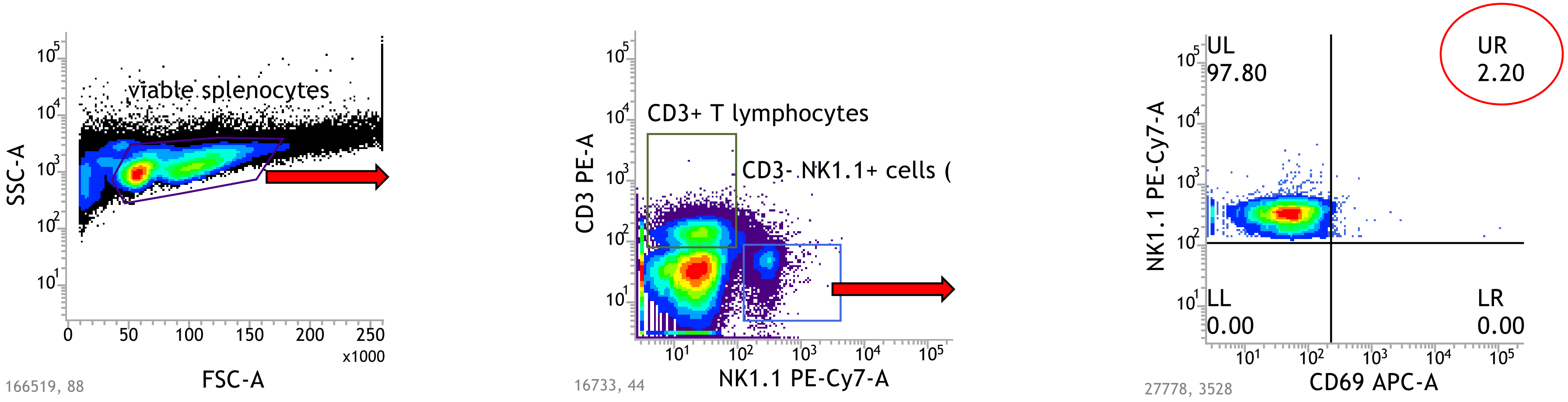

Gating Strategy for Fig.3A

SPLEEN

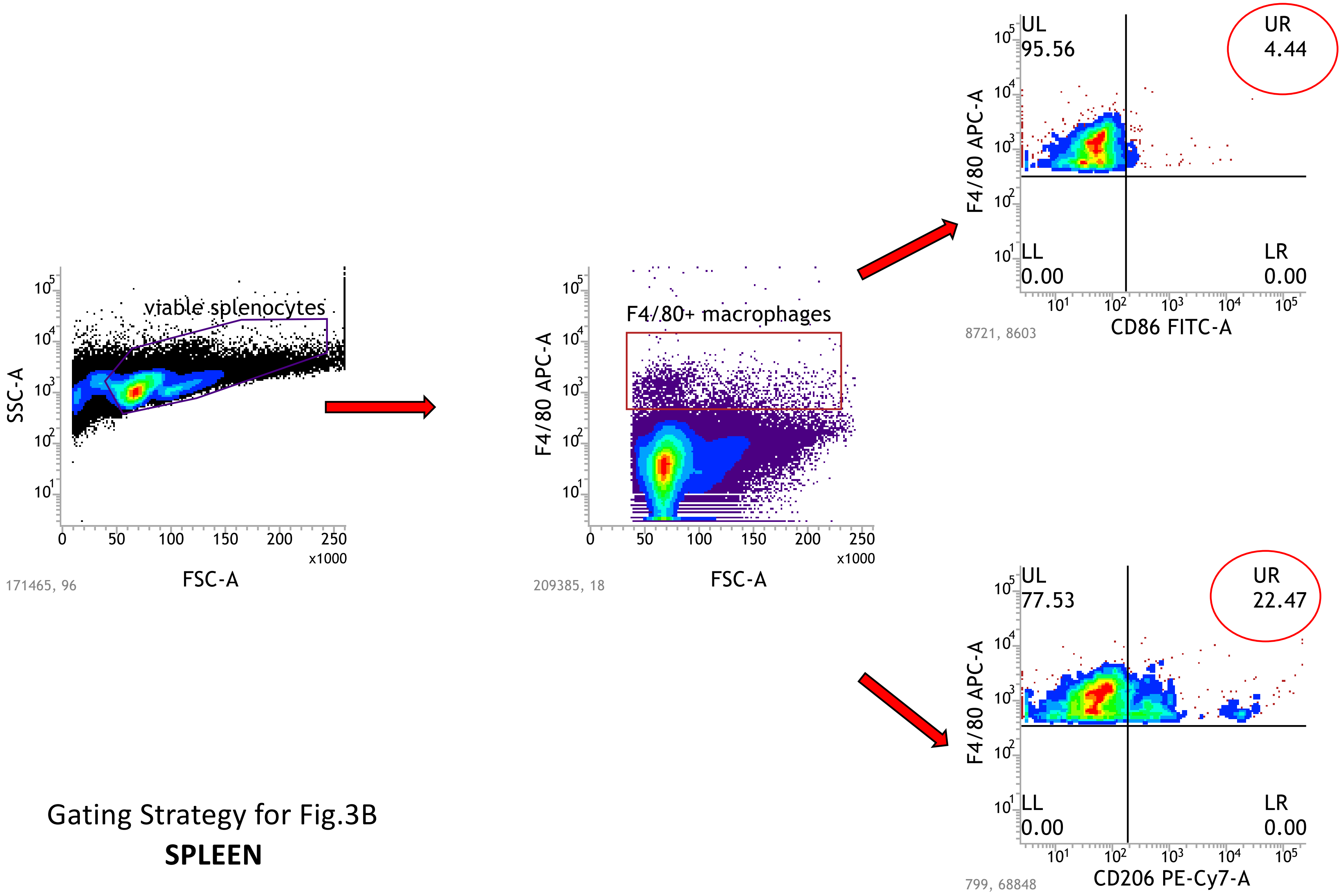

Gating Strategy for Fig.3B

SPLEEN

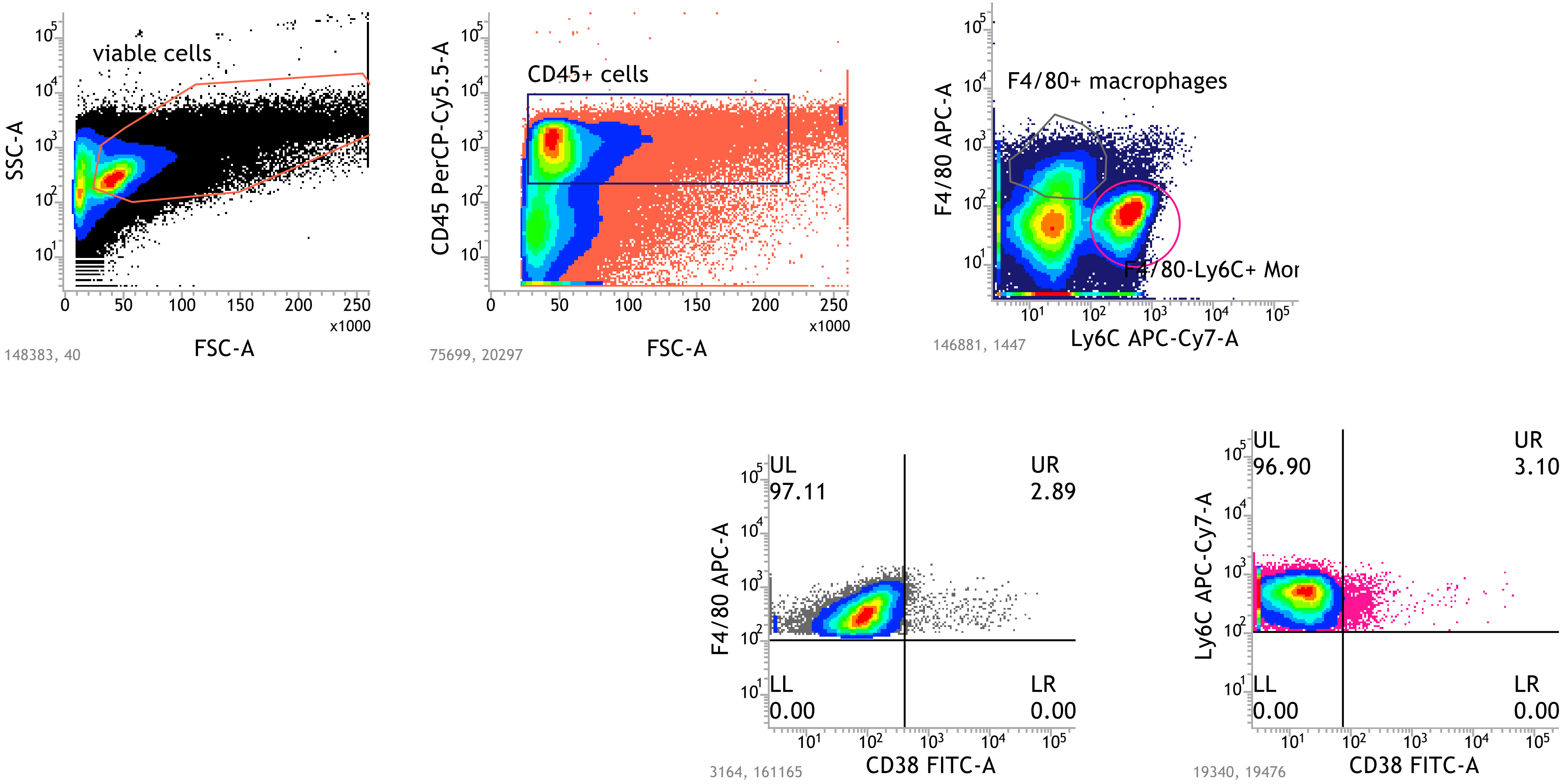

Gating Strategy for Fig.3D-E

SPLEEN

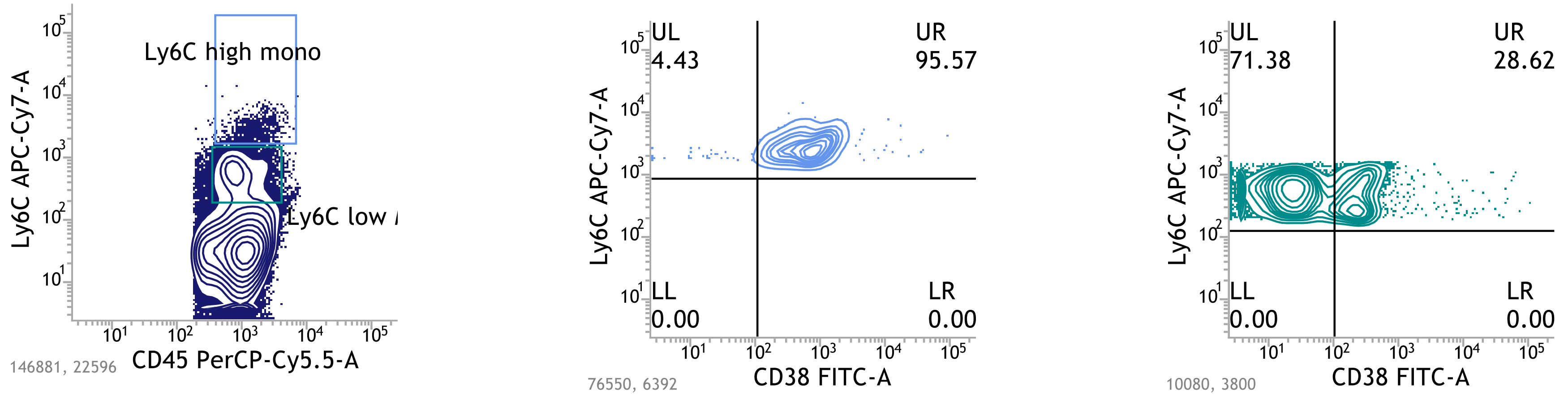

Gating Strategy for Fig.3F

SPLEEN
